# Sexual dimorphism in odontometric parameters using cone beam CT: a systematic review

**DOI:** 10.1186/s13005-023-00352-7

**Published:** 2023-03-07

**Authors:** M. A. Ajmal, Tina S. Roberts, Khaled R. Beshtawi, A. C. Raj, N. C. Sandeepa

**Affiliations:** 1 Department of Diagnostic Sciences, College of Dentistry, King Khalid University, Abha, Saudi Arabia; 2 Department of Oral and Maxillofacial Pathology, Faculty of Dentistry, University of The Western Cape, Bellville, South Africa; 3 Department of Craniofacial Biology, Faculty of Dentistry, University of The Western Cape, Bellville, South Africa; 4 Department of Oral Medicine & Radiology, Mahe Institute of Dental Sciences, Mahe, Puducherry, India

**Keywords:** Cone beam computed tomography, Odontometrics, Permanent dentition, Sexual dimorphism, Sex estimation

## Abstract

**Objective:**

To determine whether odontometric parameters using cone beam computed tomography (CBCT) would aid in sex estimation by assessing sexual dimorphism of odontometric parameters.

**Material and methods:**

The focused question was whether there is sexual dimorphism in linear and volumetric odontometric parameters when assessed using CBCT. The preferred reporting items for systematic reviews and meta-analysis (PRISMA) guidelines were followed to conduct a systematic search until June 2022 in all major databases. Data were extracted regarding the population, size of the sample, age range, teeth analyzed, linear or volumetric measurements, accuracy, and conclusion. The quality of included studies was assessed using (Quality Assessment of Diagnostic Accuracy Studies (QUADAS-2) tool.

**Results:**

Out of the 3761 studies identified, twenty-nine full-text articles were assessed for eligibility. Finally, twenty-three articles (4215 participants) that provided data on odontometrics using CBCT were included in this systematic review. The odontological sex estimation were assessed either linear measurements (*n* = 13) or volumetric measurements (*n* = 8) or both (*n* = 2). Canines were analysed in maximum number of reports (*n* = 14), followed by incisors (*n* = 11), molars(*n* = 10) and premolars(*n* = 6). Most of the reports (*n* = 18) confirmed the existence of sexual dimorphism in odontometric parameters when assessed using CBCT. No significant differences in odontometrics between the sexes were noted in some reports (*n* = 5). The accuracy of sex estimation was assessed in eight investigations, which ranged from 47.8 to 92.3%.

**Conclusions:**

Odontometrics of human permanent dentition using CBCT exhibit a certain degree of sexual dimorphism. Both linear and volumetric measurements of teeth can aid sex estimation.

**Supplementary Information:**

The online version contains supplementary material available at 10.1186/s13005-023-00352-7.

## Introduction

Defining traits of an individual are age, sex, and ethnicity. A significant component of human identity in forensics is sex recognition. Sex estimation assists in the identification of a missing person’s tentative sex, which will be utilized to perform sex-specific age estimation [1]. Age and sex determination are basic for the fabrication of the human being’s biological form. In a state of skeletonization, sex assessment provides information for the identification of cases [2]. Sex determination is essential in the diverse disciplines of forensics. It is chiefly necessary to recognize withered skeletal remnants and parts of the body. Anthropological and odontological models evaluate sex, encompassing various metric and non-metric parameters. Morphological and metric parameters of the dentition and neighbouring structures like lips, palate, mandible, and paranasal sinuses and biochemical analysis of teeth structures are used in sex estimation [1]. Various forensic and anthropological analyses extensively studied permanent human dentition to estimate sex and age. The dentition is usually well protected, no matter the state where the corpse was found or preserved [2]. Human permanent dentition resists post-mortem decomposition and has an excellent preserving capability, hence an effective source for sex estimation [3].

Digital calipers, two-dimensional digital models, graphical depictions to record measurements on dental casts, digital impression models, three-dimensional cone beam computed tomography (CBCT) models, and laser scanned models are the various straightforward techniques in odontometrics. With the evolving technology, odontological assessments in three-dimension are feasible because of three-dimensional CBCT. It is a more accurate and reliable method for measuring tooth dimensions. Storage, retrieval of images, and use of sophisticated software to perform image analysis are the possibilities that reinforce the integrity and consistency of odontometric morphometry using three-dimensional CBCT [4, 5]. Odontometrics using CBCT is par with digitalized plaster models using the digital mode, as it enables measuring tooth dimension and arch size swiftly, efficiently, consistently, and precisely [6]. CBCT is a non-invasive technique that does not need extraction or sectioning of a tooth, can be applied in mortal beings, and has the advantage over destructive methods, as they result in loss of evidence which is unacceptable [2, 7]. CBCT provides three-dimensional images of acceptable quality at a low radiation dose, it is better than two-dimensional imaging. The morphology of a tooth can be visualized from all angles without image distortion, magnification, or superimpositions. CBCT images can be viewed in multiple sections and levels [7, 8]. Visualization of the pulp chamber and anatomic variations are possible due to spatial resolution [8]. It also provides detailed information about dentition and supporting structures [9].

Sexual dimorphism reveals a set of morphologic features in the form of shape or size that differentiate a male from a female. Various species exhibit sexual dimorphism in the odontometric variables [9]. A systematic review [1] that described the features of various odontological sex estimation techniques reported an accuracy of over 80% without providing absolute discrimination. Numerous studies attempted to recognize the distinction between sexes by evaluating human teeth dimensions in different populations, with inconsistent findings [10]. Taking into consideration of several dental anatomical studies in literature and the variation in their results, diversity observed in tooth dimensions is of fundamental importance; CBCT is a new modality to study anatomical diversity that has a bearing on the future of dental anatomy and forensic odontology [10]. The present analysis aimed to systematically review reports assessing odontometric sex estimation using CBCT. The research question was whether there is sexual dimorphism in the odontometric parameters when assessed using CBCT.

## Methods and material

This investigation was performed by the guidelines of the Preferred Reporting Items for Systematic reviews and Meta-analyse (PRISMA) statement (for diagnostic test accuracy). The review protocol was prospectively registered in PROSPERO (Registration number: CRD42022335735) where it can be accessed online at https://www.crd.york.ac.uk/prospero/display_record.php?ID=CRD42022335735.

### Focused question and study eligibility

The focused question for this review was- Is there sexual dimorphism in odontometric parameters of the human permanent teeth when assessed using CBCT?

1. Whether there is sexual dimorphism in linear odontometric parameters using CBCT?
2. Do volumetric odontometric parameters show sexual dimorphism when assessed using CBCT?

The framework that was used to formulate the research question was based on population, index test, reference test and diagnosis of interest – [population (CBCT images from human subjects), index test (linear/volumetric odontometric methods using CBCT), reference test (reference standard, such as expert’s judgment and medical record) and diagnosis of interest (sexual dimorphism of odontometric parameters)] [11].

### Inclusion criteria for the studies were as follows

1) clinical studies carried out in various populations that investigated different odontometric parameters of permanent human dentition using CBCT, 2) no restrictions on ethnicity, 3) studies that compared male and female subjects, 4) studies that compared index tests as per eligibility criteria to the study’s reference standard, 5) analysis of linear and volumetric measurement of permanent teeth, odontometric population traits, and sexual dimorphism from cross-sectional investigations and studies that report diagnostic accuracy.

### Exclusion criteria for the studies were as follows

1) studies using casts, extracted teeth, non-human teeth, skeletal remains, direct intraoral assessment, intraoral photography, x-ray, computed tomography (CT) scan, and micro CT scan for sex estimation, 2) non-metric features of teeth, 3) techniques based on cephalometric, sinuses measurements, cheiloscopy, palatal rugoscopy, and biochemical analysis for sex estimation, 4) populations with known systemic diseases, syndromes, or other pathologies affecting permanent teeth and studies with small samples (*n* < 10). In vitro studies, case reports, case series, books, book chapters, conference papers, editorials, letters to the editor, and systematic reviews were also excluded.

### Study search strategy and process

The PRISMA protocol was followed to conduct and report this systematic review (PRISMA (Preferred Reporting Items for Systematic Reviews and Meta-Analyses) statement. Studies from 01 Jan 2000 till date (30 June 2022) that met the eligibility criteria were included in this review. The search was carried out on all major databases, such as PubMed, Scopus, Web of Science, Google Scholar, and Cochrane, to identify reports of odontometric analysis restricted to permanent human dentition using CBCT.

The search was conducted based on the research question’s main three concepts (CBCT, odontometrics, and sexual dimorphism). The literature database was searched using MeSH terms, keywords, and other free terms related to CBCT (“CBCT” OR “cone beam computed tomography), odontometrics (“odontometry” OR “teeth” OR “volumetric assessment” OR “volume measurement” OR “pulp cavity volume” OR “buccolingual dimension“) and sexual dimorphism (“sexual dimorphism” OR “gender assessment” OR “sex assessment” OR “sex determination” OR “gender determination” OR “gender prediction” OR “sex estimation”) for identifying relevant publication up to 30 June 2022. The details are given in additional file 1 which depicts the PubMed search strategy.

In addition, references to relevant studies and manual searching also were done for other potentially appropriate publications.

### Data extraction and outcome of interest

The inclusion criteria were refined by piloting the study selection process. Data extraction was done from the selected studies and tabulated using excel sheets.

Two blinded reviewers (AJ and RA) independently screened all the titles and abstracts identified through electronic and manual searches. Studies that did not fulfill the inclusion criteria were excluded. Next, full-text papers that fulfilled the eligibility criteria were identified and included in the review. Disagreements regarding the inclusion or exclusion of studies were resolved by discussion with a third reviewer (SN).

Data were collected regarding the linear and/or volumetric measurement of each study. This included author, year, country of origin, age of the participants, sample size, type of measurement (whether linear or volumetric), parameters assessed in the odontometric methods, tooth/teeth analyzed, whether sexual dimorphism present (yes/no) and the accuracy of sex estimation. The studies were classified based on the type of measurements and type of teeth assessed using CBCT.

### Quality analysis

The quality of included studies was assessed using (Quality Assessment of Diagnostic Accuracy Studies (QUADAS-2) tool [12]. To measure the quality of each study, four key domains in two categories (risk of bias and applicability concerns) were assessed. The risk of bias was assessed under four domains (patient selection, index test, reference standard, and flow and timing) with relevant signaling questions. Similarly, applicability concerns were assessed based on patient selection, index test, and reference standard. The responses were color-coded and marked to indicate high risk, low risk, and unclear.

## Results

### Study selection

A total of 3761 records were identified through various databases and hand searching. After removing duplicates, 3664 studies underwent title and abstract screening where 3635 studies were excluded. Twenty-nine full-text articles were assessed for eligibility, out of which 6 were excluded for various reasons (Table 1).

**Table 1:** Excluded studies with reason

Sl No	Author	Title	Reason for exclusion
1	Tardivo et al. 2011 [13]	Three-dimensional Modeling of the Various Volumes of Canines to Determine Age and Sex: A Preliminary Study	The sample consisted of 58 dental computerized tomography (CT) scans.
2	Tardivo et al. 2015 [14]	Gender Determination of Adult Individuals by Three-Dimensional Modeling of Canines	The types of CT scans selected for the sample were brain scans, brain angioscans, dental scans, and ENT scans.
3	García-Campos et al. 2018 [15]	Modern humans sex estimation through dental tissue patterns of maxillary canines	The sample was selected from the anthropological collections housed Madrid (Spain), Pretoria (South Africa) and Sudan. The specimens were scanned in three facilities: Microtomographic system housed in the Microscopy Laboratory.
4	García-Campos et al. 2018 [16]	Contribution of dental tissues to sex determination in modern human populations	Teeth included in this study were selected from anthropological collections from Spain, South Africa and Sudan. One part of the sample was scanned using a microtomographic system housed in the Microscopy Laboratory.
5	Krenn et al. 2019 [17]	Variation of 3D outer and inner crown morphology in modern human mandibular premolars	MicroCT datasets Premolars specimens with morphological variation within and between geographically diverse modern human groups from five continents using Geometric morphometric analysis
6	Sorenti et al. 2019 [18]	Sexual dimorphism of dental tissues in modern human mandibular molars	Variables were assessed from two-dimensional (2D) mesial planes of section obtained from microtomographic scans. Spanish anthropological collection of teeth used for this study was scanned using a CTPMlab micro-CT.

**Fig. 1:**
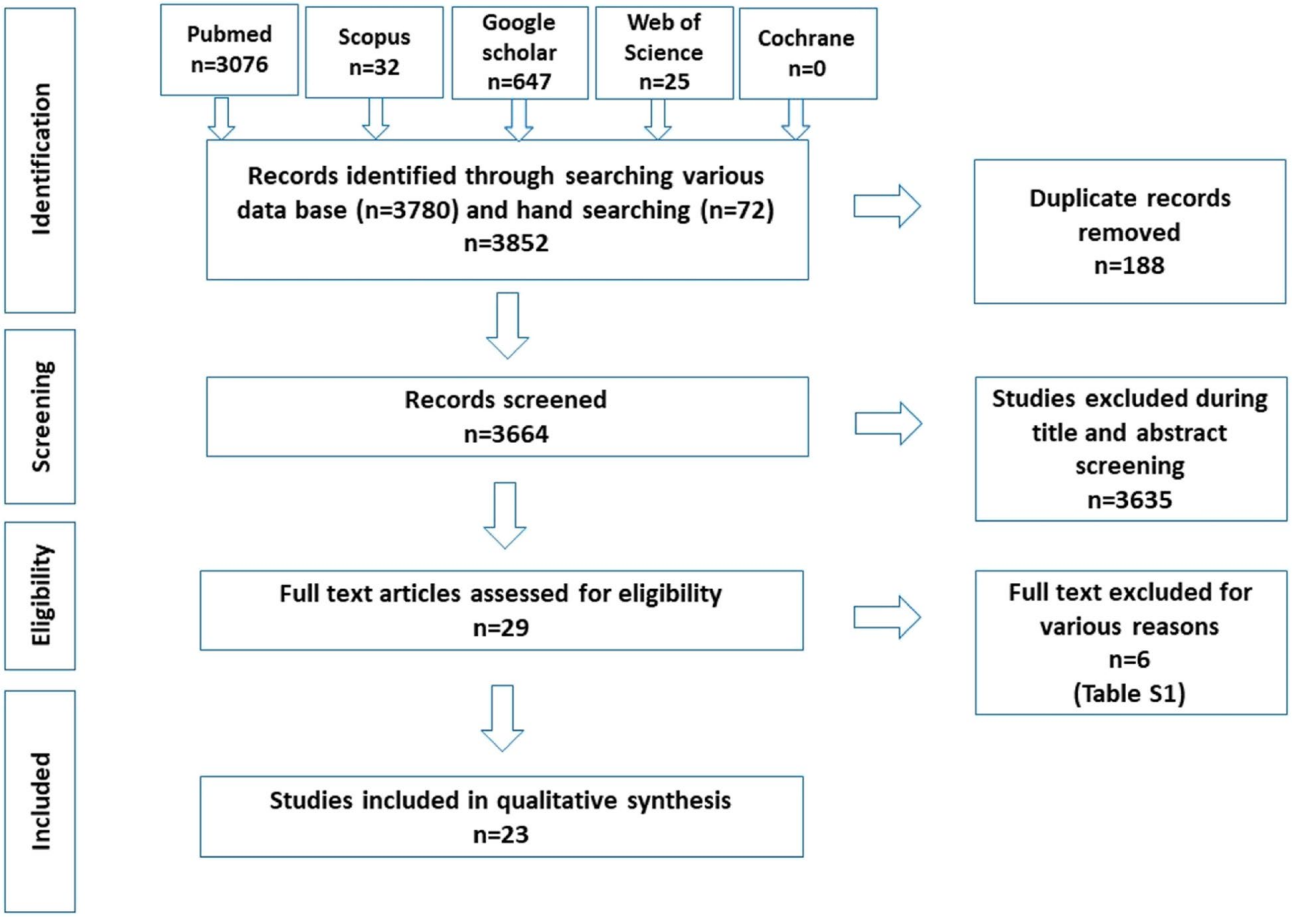
Summary of the systematic review workflow using PRISMA chart

Twenty-three articles were finally included in the systematic review (Fig. 1).

### Studies characteristics

Of the twenty-three studies, four were investigated in Brazil [2, 3, 19, 20], four in India [7, 8, 21, 22], three in Iran [23, 24, 25], three in Turkey [26, 27, 28], two in Saudi [4, 5], one each in Poland [29], Portugal [30], Egypt [9], Ukraine [31], Italy [32], Spain [33] and Malaysia [6].

These 23 studies involved 4215 participants (2103 males and 2112 females), and the sample sizes ranged from 53 to 1190 (Table 2).

**Table 2:** Characteristics of the included studies in the systematic review

Serial No	Author & Year Country	Age of the participants	Sample size or CBCT Images (female/male)	Linear/ Volumetric	CBCT features (Device type, voxel resolution,slice type)	Parameters assessed in the odontometric methods	Teeth analyzed	Sexual dimorphism present (yes/no)	Accuracy of sex estimation
1	Paknahad et al. 2022 [24] Iran	15–25 years; (21.28 ± 2.47 yrs)	200 CBCT images of 200 subjects; 100 females and 100 males	Linear	New Tom VGi; scan time, 8.9 seconds; 5 mA; 19 mAs; 120 kV; coronal slices reconstructed with a slice thickness of 0.3 and a slice interval of 1 mm	The roof, floor, height of the pulp chamber, mesial and distal enamel thickness, dentin thickness at the height of contour and crown length	Maxillary and mandibular first molar teeth	Yes	Overall 80.5% accuracy in sex estimation when first molars were used.
2	Bansal et al. 2022 [7] India	20–65 years	60 CBCT images of 60 subjects; 29 females and 31 males	Linear	Sirona digital imaging system with 70–74 kV, 10 mA, and 10.8 s, with a field of view (FOV) 8 cm × 8 cm, and voxel size of 160 μm [3]; sliced into three dimensions; sagittal, coronal and axial sections of central incisor	Pulp-to-tooth area ratio	Maxillary canines and central incisors	No	Not stated
3	Warnecki et al. 2022 [29] Poland	17–35 years	92 CBCT images of 92 subjects;43 females & 49 males	Linear	Carestream CS 8200 CS 3D, Carestream Health, Rochester, NY, USA; length of each tooth was measured separately in the sagittal section with 3D Slicer	Lateral skull radiograph; SNA, SNB and ANB angles; presence (or absence) of asymmetry in facial features; a shift of the chin greater than 5 mm relative to the facial midline; length of each tooth	Single rooted and multi-rooted teeth	Yes	Not stated
4	Yagci et al. 2021 [28] Turkey	20.88 ± 2.48 years	560 CBCT images of 101 subjects;57 females and 44 males	Linear	NewTom 5G, QR, (Verona, Italy); scanning time 14–18 seconds; collimation height 13 cm; exposure time 3.6 seconds; and voxel sizes were 50 μm)	Enamel thickness; proximal areas of maxillary and mandibular incisors, canines, and premolars.	Maxillary and mandibular incisors, canines and premolars	Yes	Not stated
5	Esmaeilyfard et al. 2021 [23] Iran	15–25 years; Mean age of 21.28 ± 2.47 years	485 CBCT images of 485 subjects;240 females and 245 males	Linear	New Tom VGi; scan time 8.9 s, 5 mA, 19 mAs, 120 kV; corrected sagittal and coronal slices were reconstructed with a slice thickness of 0.3 and a slice interval of 1 mm	Roof, floor, and height of the pulp chamber, enamel and dentin thickness at the height of contour, tooth width, crown length in both buccolingual and mesiodistal aspects	Permanent first molars	Yes	Average classification accuracy was 92.31%.
6	De Koninck et al. 2021 [30] Portugal	18–60 years	58 CBCT of 58 subjects; 27 females and 31 males	Linear	ProMax 3D Plus device (Planmeca, Finland); voxel size of 0.2 mm and 0.15 mm and the same energy parameters (90 kV and 10 mA)	Maximum buccolingual dimension of the cervical root measured in the midsagittal plane	Mandibular canine	Yes	79% accuracy in estimating the sex.
7	Denny et al. 2021 [8] India	15–30 years	100 CBCT images of 100 subjects; 56 females and 44 males	Linear +Volumetric	Promax 3D, Mid version (Planmeca Oy., Helsinki, Finland)	Height of the crown, height of the coronal pulp cavity, tooth – coronal index	Four mandibular molars	No	Not stated
8	Fauzi 2021 [21] India	No Mention	100 CBCT images of 100 subjects; 56 females and 44 males	Linear	CBCT	Mesiodistal width	Mandibular central incisor	Yes	Not stated
9	Fardim et al. 2021 [3] Brazil	20–65 years	1190 CBCT image of 1190 subjects;606 females and 584 males	Volumetric	I-CAT Next Generation (Imaging Sciences International, HA, PA, EUA); voxel of 0.25 mm; Axial, coronal and sagittal image slices.	Pulp chamber volume	Mandibular canines, pre-molars and molars	Yes	Not stated
10	Topbas and Okkesim 2021 [27] Turkey	12–69 years	332 CBCT images of 332 subjects; 162 females and 170 males	Volumetric	Planmeca 3D Mid (Planmeca, Helsinki, Finland) device with a voxel size of 200 μm and using 85 kVp, 10 mAs and 14 s scanning time; field of view was 15 × 9 cm.; voxel size: a 0.2 mm^3^	Pulp volume, tooth volume	Maxillary or mandibular molar tooth	Yes	76.6% in estimating female sex and 56.3% accuracy in estimating male sex.
11	Salam et al., 2021 [9] Eygpt	18–25 years	100 CBCT images of 100 subjects; 50 females and 50 males	Linear	Planmeca, Promax 3D Max, Finland; all scans were output with 512 X 512 pixels per slice and 8 bits per pixel; voxel size: 0.16 mm^3^.	Crown width and height, enamel thickness, arch depth, and width.	Mandibular permanent canines and first molars	Yes	Not stated
12	Issrani et al. 2020 [5] Saudi Arabia	17–35 years	400 CBCT images of 100 subjects; Equal males and females	Volumetric	3D CBCT	Mesio-distal width; inter-canine distance; Mandibular canine index	Mandibular canines	No	47.8% accuracy in estimating sex.
13	Shalakizadeh et al., 2020 [25] Iran	20–50 years	133 CBCT of 266 subjects; 156 females and 110 males	Linear	Newtom VGi CBCT unit (Verona, Italy). 1536 × 1920 pixels, a pixel size of 127 × 127, a pixel depth of 14 bits, a rotation of 360 degrees, a scan time of 18 s and a kVp of 110; slice thickness of 1 mm at 0.25 mm intervals	Mean length of canine teeth	Maxillary and mandibular canine	Yes	Not stated
14	Manhaes-Caldas et al. 2019 [19] Brazil	8–36 years	128 CBCT images of 128 subjects; 64 females and 64 males	Volumetric	Picasso Trio unit (Vatech, Hwaseong, South Korea), adjusted at 80 kVp, 3.7 mA and a voxel size of 0.2 mm; 3D Slicer software, version 4.8 (sagittal and coronal reconstruction).	Total volume of the dental crown; Image-based volumetric assessment by manual segmentation	Upper central incisors, upper and lower canines, and lower lateral incisors	Yes	83.7% accuracy in estimating sex.
15	Alam et al. 2019 [4] Saudi Arabia	20–45 years	252 CBCT Images;252 subjects; 93 females and 159 males	Linear + Volumetric	3D CBCT	Tooth size from 2nd molar to 2nd molar of maxillary and mandibular arch	Full dentition (except 3rd molars)	No	Not stated
16	Andrade et al. 2019 [2] Brazil	13–70 years	116 CBCT images of 116 subjects sample were homogenous in terms of age and sex; total of 232 teeth	Volumetric	Kodak K9500R scanner (Carestream Health, Rochester, RY); voxel size of 0.2 mm^3^ and 0.3 mm3 and the same energy parameters (90 kVp and 10 mA); field of view (15 × 9 cm and 20 × 18 cm).	Pulp volumes	Upper central incisors and canines	Yes	High accuracy of sex estimation
17	Rahman and Ramakrishnan 2019 [22] India	18–40 years	50 CBCT images of 50 subjects;25 females and 25 males	Linear	CBCT	Mesio-distal width at apical third, middle third, and cervical third region.	Maxillary central incisors	Yes	Not stated
18	Kavas and Tumen 2019 [26] Turkey	7–18 years	80 CBCT images of 80 subjects; 40 females & 40 males; 160 M	Volumetric	i-CAT (Imaging Sciences International, Hatfield, PA, USA); voxel size: 0,3 mm; 3D Slicer	Pulp chamber volume	Mandibular and maxillary first molars	Yes	Not stated
19	Marchenko et al. 2017 [31] Ukraine	Adolescents	77 CBCT images of 77 subjects; 42 females and 35 males; mesocephalic boys - 16, boys brachycephalic – 19, mesocephalic girls – 16, brachycephalic girls– 26	Linear	Veraviewepocs 3D, Morita (Japan); 3D image - cylinder 8x8cm - thickness 0,2/0,125 mm, dose of radiation 0,11–0,48 mSv, voltage and amperage 60-90 kV/2-10 mA	Vestibular-oral and mesio-distal projection length of the root; Length of roots of incisors and canines of the upper and lower jaw	Upper and lower Incisors and canines	Yes	Not stated
20	De Angelis et al. 2015 [32] Italy	15–83 years	87 CBCT images of 87 subjects; 46 females and 41males	Volumetric	i-Cat Next Generation (Imaging Sciences International, Hatfield, Pa); voxel size: 0.4 mm; scan time: 8.9 s; scan width: 23.2 cm; scan height: 17 cm	Dental volume; mean dental volume, minimum dental volume; maximum dental volume mean pulp chamber volume, minimum pulp chamber volume; maximum pulp chamber volume; mean percentage ratio between pulp chamber and tooth.	Upper Canines	Yes	Overall 80.5% accuracy in estimating the sex.
21	Porto et al.2015 [20] Brazil	22–70 years	72 CBCT images of 118 subjects; 60 females and 58 males	Volumetric	i-CAT® Next Generation (Cone Beam 3-D Dental Imaging System e Imaging Sciences International, Hat!eld, PA/USA); (voxel-size) of 0.25 mm; stores only the axial images	Pulp cavity volume, hard tissue volume, tooth volume and pulp cavity/tooth volume ratio	Upper central incisors	No	Not stated
22	Llena et al. 2014 [33] Spain	Mean age of 45.26 years	70 CBCT images of 70 subjects;31 females and 39 males;126 premolars	Linear	Kodak 9000 3D unit (Carestream Dental, Atlanta, GA, USA); 70 kV, 10 mA, and 10.8 s; μm; voxel size: 76 μm	Tooth position in the arch, number of roots, length of the tooth and root, number of canals, canal system configuration; Distance from the cemento-enamel junction to the canal bifurcation and canal reunification, number of foramina, root curvature, angle location, and distance of the angle vertex to the apex	Lower premolars	Yes	Not stated
23	Alam et al. 2014 [6] Malaysia	16–35 years	53 CBCT images of 53 subjects; 21 females and 32 males	Linear	Plameca Promax 3D (Helsinki, Finland); transaxial and sagittal slices (1 mm)	Tooth size, arch length, arch perimeter, inter-canine, inter-first premolar, inter-second premolar and inter-molar widths	Incisors, canines, premolars, 1st molar; arch length & arch perimeter	Yes	Not stated

The odontometric sex estimation by CBCT used linear measurement in 13 studies, volumetric measurement in 8 studies, and both these measurements in 2 studies. 92% (12/13) of studies on linear measurement could estimate sex correctly, while 75% (6/8) of studies on volumetric measurement favoured sex estimation. Teeth measurements for sex estimation were mainly performed on CBCT images using various image analysis software such as ITK-SNAP and Romexis. Sexual dimorphism in odontometric parameters was observed in all teeth across the populations, mostly in canines followed by incisors and molars.

Of the teeth in the permanent dentition using CBCT images, canines were analyzed in a maximum number of studies (*n* = 14), followed by incisors (*n* = 11), molars (*n* = 10), and premolars (*n* = 6). More than one type of teeth was analyzed in ten reports**.** All types of teeth were analyzed in three reports. Most of the reports found a positive response that odontometrics could aid in sex estimation (*n* = 18). 78% of reports confirmed the existence of sexual dimorphism in odontometric parameters. The accuracy of sex estimation was assessed in eight investigations, which ranged from 47.8 to 92.3%. No significant differences in tooth dimensions between the sexes were noted in five reports **(**Table 2**)**.

Of the 14 studies in canines, 71% (10/14) supported the existence of sexual dimorphism in odontometrics of permanent canines. Tooth size, tooth length, dental volume, the volume of the pulp chamber, mandibular canine crown width and length, inter-canine width, buccolingual root dimension of mandibular canine, and mandibular canine index are primary odontometric parameters that made a sex difference. A significant sex difference was not appreciated in root length, enamel thickness, and pulp tooth ratio. Even in incisors, root length and pulp tooth ratio did show any significant difference between the sexes. Of eleven studies assessing CBCT images of incisors, 73% (8/11) of studies supported the presence of sex difference in linear or volumetric measurement of permanent incisors.

Among ten studies assessing various odontometric parameters of permanent molars, 60% (6/10) reports demonstrated the existing sex difference in odontometrics of molars. The most dominant variables which made a difference include crown height, width, intermolar distance, dentin thickness, volume ratio, and total pulp chamber volume. Out of six studies evaluating the premolars, 67% (4/6) reports showed sexual dimorphism; length of tooth and roots, mesiodistal crown width, and enamel thickness are the odontometric variables that made the difference.

ITK-SNAP software and Romexis software were common tools used in many reports. In four studies, there is no mention (non-specification) of the software used to analyze the CBCT images [9, 21, 23, 31]. Five studies did not find sexual dimorphism through odontometrics [4, 5, 7, 8, 20], while in two reports, statistical analysis details were minimal/ not specified [22, 32]. Only four studies mentioned sampling by randomization, and the remaining nineteen selected studies used convenience sampling [4, 5, 24, 26].

### Study quality assessment

The QUADAS 2 scoring showed an overall low risk of bias **(**Table 3**)**. Fifteen articles scored low-risk scores for all seven criteria. Four studies showed low risk for most of the parameters with few unclear parameters. The remaining four studies had unclear risks.

**Table 3:** QUADAS-2 assessment of the included studies

Author	Risk of bias (High/Low/Unclear)				Applicability concerns (High/Low/Unclear)		
	Patient selection	Index test	Reference standard	Flow and timing	Patient selection	Index test	Reference standard
Paknahad et al. 2022 [24]	**+**	**+**	**+**	**+**	**+**	**+**	+
Bansal et al. 2022 [7]	**?**	+	+	+	**?**	+	+
Warnecki et al. 2022 [29]	**?**	+	+	+	**?**	+	+
Yagci et al. 2021 [28]	+	+	+	+	+	+	+
Esmaeilyfard et al. 2021 [23]	+	+	+	+	+	+	+
De Koninck et al. 2021 [30]	+	+	+	+	+	+	+
Denny et al. 2021 [8]	**?**	+	+	+	**?**	+	+
Fauzi 2021 [21]	**?**	**?**	+	+	**?**	**?**	+
Fardim et al. 2021 [3]	**?**	**?**	**?**	+	**?**	**?**	**?**
Topbas and Okkesim 2021 [27]	+	+	+	+	**+**	**+**	**+**
Salam et al., 2021 [9]	**–**	+	+	+	**–**	**+**	**+**
Issrani et al. 2020 [5]	+	+	+	+	**+**	**+**	**+**
Shalakizadeh et al., 2020 [25]	+	+	+	+	**+**	**+**	**+**
Manhaes-Caldas et al. 2019 [19]	**–**	+	+	+	**–**	**+**	**+**
Alam et al. 2019 [4]	+	+	+	+	**+**	**+**	**+**
Andrade et al. 2019 [2]	+	+	+	+	**+**	**+**	**+**
Rahman&Ramakrishnan 2019 [22]	**?**	+	+	+	**?**	**+**	**+**
Kavas ans Tumen 2019 [26]	+	+	+	+	**+**	**+**	**+**
Marchenko et al. 2017 [31]	+	+	+	+	**+**	**+**	**+**
De Angelis et al. 2015 [32]	+	+	+	+	**+**	**+**	**+**
Porto et al. 2015 [20]	**+**	+	+	+	**+**	**+**	**?**
Llena et al. 2014 [33]	**?**	**+**	**+**	+	**?**	**+**	**+**
Alam et al. 2014 [6]	**+**	**+**	**+**	+	**+**	**+**	**+**

High risk: -; Low risk: +; Unclear risk:?

In-depth assessment regarding sexual dimorphism was difficult due to the small sample size in few reports, especially if the investigation intended to assess differences between the sexes [6, 22, 30]. Hence, it was regarded that these reports showed a risk of bias for the estimation of differences in the linear or volumetric measurements of permanent teeth. Only two studies mention power analysis used for sample size estimation [5, 27]. The intra-class correlation coefficient was applied in most reports to analyze intra-observer and inter-observer agreements. Poorly explained calibration features in the methodology or a lack of complete information assessed the risk of bias as ‘unclear’ in a few studies.

## Discussion

This systematic review aimed to determine whether there is sexual dimorphism in the odontometric parameters when assessed using CBCT. Of these published 23 studies, eighteen studies found a positive response that odontometric measurements could aid in sex estimation. The majority found sexual dimorphism in various odontometrics of permanent canines, especially with mandibular canines.

A recent systematic review on tooth crown mesiodistal measurements for estimating sexual dimorphism across a span of different people confirmed that canines reflect the greatest sexual dimorphism [10]. Several investigations found canines and first molars to be the most common teeth with a lot of morphological diversity between sexes [10, 34]. In the present analysis, sexual dimorphism was observed in all teeth across various populations, mostly in canines, followed by incisors, premolars, and molars. Tooth formation and development are controlled by sex-related genes, and structures of human permanent dentition exhibit sex differences. Accordingly, forensic researchers have evolved several methods to distinguish males from females. But still, experts choose canines for sex estimation [27].

Of the teeth in the permanent dentition using CBCT images, canines were analyzed in fourteen studies out of twenty-three studies. Tooth size or dimension is the most frequently assessed odontometric variable for sex estimation. The tooth size of the maxillary and mandibular canine displays the largest variation of sexual dimorphism [6]. Prolonged amelogenesis in males results in differences in enamel thickness between the sexes and, consequently, greater dimensions of canines in males than in females [35]. The ‘Y’ chromosome promotes mitotic activity in tooth germs and controls growth by enhancing amelogenesis and dentinogenesis, consequently greater dentin thickness in males [36]. In contrast, the ‘X’ chromosome controls only the enamel growth. This could explain the variation in size [36].

Few investigators consider that the sexual dimorphism of mesiodistal width is because of dentin deposition, which is in excess in men than in women. On the contrary, there is no difference in enamel thickness [32]. Few researchers contemplate the variation in the level of sex hormones in the course of tooth formation could influence tooth dimensions [9]. Parameters like crown width and length of mandibular canine and inter-canine width show highly significant sex differences [9]. The mesiodistal width of mandibular canines revealed statistically significant sexual dimorphism [5]. In permanent dentition, mandibular canines are known to show the greatest sex dimorphism; hence, it has become the tooth of choice for sex estimation studies. It has been considered that the mesiodistal width of mandibular canines is the simpler method for sex prediction with a better rate of accuracy [37]. A significant difference in the length of canines in both sexes and both jaws also have been reported [25].

Average tooth length is greater in men than in women [29]. In both women and men, the longest teeth turned out to be canines. The buccolingual root size on the mandibular canine also revealed significant differences between the sexes [30]. The average values of the canine pulp chamber were larger for males compared to females [3]. Dental volume showed a significant difference between sexes [32]. The dental volume observed a significant difference between the sexes, while different finding was noted for the volume of the pulp chamber and the ratio between the pulp chamber and dental volume. The lack of sexual dimorphism in the pulp chamber quantification is probably due to the effect of age on the pulp dimension [32]. Volumetric accuracy of the maxillary canine and mandibular canine for sex estimation were 74.4 and 79.5%, and the combined analysis of the maxillary and mandibular canines allowed an average accuracy of 83.7% [19].

While reviewing the literature on odontometric assessment of permanent incisors using CBCT for estimation, it was observed that the largest variation in the tooth dimension was found in the maxillary lateral, second premolars, and mandibular lateral incisors in men, whereas the maxillary canine and mandibular incisors in the women [6]. Males show a greater mean mesiodistal dimension of central incisors than females [22]. The mesiodistal dimension of both maxillary central incisors is significantly different in males compared to those in females. Though the form and shape of tooth structure are similar in both sexes, the size might differ, as tooth dimension is influenced by genetic, cultural, racial, and environmental factors [38].

Warnecki et al., 2021 found that mean tooth length is greater in men than in women [29]. In both men and women, the smallest difference in tooth length between women and men was found for the central incisors. In the same study, significant differences were found in tooth length between the sexes when evaluating the literature on odontometry of premolars using CBCT [29]. Males had significantly lengthy mandibular premolars than females. Llena et al., 2014 noted that the mean length of teeth and roots was significantly longer in males than in females [33]. Analysis of extracted premolars in the Jordanian population showed similar findings [39].

In the review of molar morphometry using CBCT in sex estimation, Paknahad et al., 2022 found that accuracy of sex estimation of the mandibular and maxillary first molar tooth was 84 and 77%, respectively [24]. The mesiodistal variables were more accurate in sexual dimorphism than the buccolingual ones. For sexual dimorphism, the most dominant variables for maxillary and mandibular first molar teeth were crown height and dentin thickness. Tobpas et al., 2021 found that sex was predicted by maxillary first molar volume ratio in 76.6% of females and 56.3% of males; it was observed that maxillary first molar tooth volume ratio provided more precise results in females’ sex estimation [27]. Salam et al., 2021 observed significant sex differences in mandibular first molar crown width and length and inter-molar width [9]. Chandler et al., 2003 found a significant difference between sexes and found that permanent first molar teeth pulps exhibit sexual dimorphism [40]. Pulp dimensions of the permanent first molar tooth in men are larger than that in women. Molars are the first permanent teeth to erupt in the mouth; hence, they are easily accessible for sex estimation at an early age when compared with other permanent teeth. It has the edge over canines, which have a greater propensity of being impacted and thus are not accessible for odontometric analysis [9].

The accuracy of sex estimation was assessed in eight investigations, which ranged from 47.8–92.3%. An accuracy of 100% for canine dimensions was reported and accepted that a small sample was responsible for the inflation of accuracy [41]. Considering the diversity in methodology, ethnicity of population and sample size, and age range, comparison of accuracies was not easy. Biochemical methods, DNA-PCR, fluorescent microscopy of the freshly extracted tooth, and analysis of Barr body provided 100% accuracy. Odontometrics on casts, skeletal remains, and pulp/tooth volume ratios on CBCT reported isolated accuracies of a cent percent [1]. Even the cascade of techniques reported a range of accuracy similar to individual methods.

Jaysinghe et al. 2022 found significant difference in all maxillary arch parameters using CBCT such as width of the alveolar ridge at the canine, first molar and second molar and the distance of the arch at the inter canine distance and junction between the hard and soft palate when assessed between the different genders [42].

There is some evidence of reverse dimorphism too in the literature related to dental structures. For example- Fernee et al. 2021 found larger surface areas and volumes of enamel and crown volumes in females unlike in the case of dentine and root, which were larger in males [43]. This was particularly seen in the case of upper canine. But none of these studies used CBCT for estimation. Further research is needed to establish the potential use of oral tissues for sex estimation in humans.

### Strengths and limitations

Most of the studies included had a nearly equal distribution of both sexes. The majority of the participants were young adults between the second the fifth decade. This ensured that the dentition is less worn off in the case of odontometrics, morphologically unchanged, and shows adequate skeletal growth and development. The added benefits of CBCT are that it enables the assessment of a subject’s tooth dimensions and arch size. The analysis can be done directly using digitized images, and record maintenance is not an issue. CBCT imaging provides a platform to make linear and/or volumetric measurements of dentition. It is a better system to archive patient details and easy access to the records that will help in analyzing tooth dimensions at convenience directly from images using various software.

The present review did not separate studies based on the software used for taking measurements directly from CBCT images or software used to reconstruct the models to make odontometric analyses. As the difference between the two modes is unknown/ unclear, this could contribute to a risk of bias. Potential problems regarding sex estimation based on odontometric analysis using CBCT are diversity with respect to sample size, class of teeth assessed, odontometric parameters analyzed, and software used to analyze.

The reasons for not subjecting the cumulative data to meta-analysis are lack of uniformity in the data and specific protocol followed in these studies. The present analysis excluded studies related to anthropological skeletal remnants because the dimensions of the dentition might be withered; similarly, intrinsic and extrinsic variables are particular to specific populations. Hence, the results focused on adolescents and adults with permanent dentition.

The reasons for not subjecting the cumulative data to meta-analysis are lack of uniformity in the data and specific protocol followed in these studies. The present review did not separate studies based on the software used for taking measurements directly from CBCT images or software used to reconstruct the models to make odontometric analyses. As the difference between the two modes is unknown/ unclear, this could contribute to a risk of bias. Potential problems regarding sex estimation based on odontometric analysis using CBCT are diversity with respect to sample size, class of teeth assessed, odontometric parameters analyzed, and software used to analyze. Although the risk of bias assessment using The QUADAS 2 chart showed an overall low risk, few studies showed some unclear parameters and unclear risks. Small sample sizes, lack of power analysis in many studies, and insufficient explanation for calibrations are some of the major reasons. Grading of recommendations assessment, development and evaluation (GRADE) assessment was not performed in this systematic review to rate the certainty of evidences which is a limitation of the review.

Most recent reports about dental anatomy are with three-dimensional technologies, with exceedingly accurate results compared to previous studies, which focused on other methods of sex estimation using casts, extracted teeth, non-human teeth, skeletal remains, direct intraoral assessment, intraoral photography, and x-ray. Even with non-metric methods of sex estimation, some important, relevant information would have been lost. Therefore, a comparison of results becomes difficult to interpret. Included studies in this review exhibit the differences in the data sets, methodology, statistics applied, and results obtained. The consensus was challenging to achieve due to the diversity in linear and volumetric measurements applied in each class of teeth. The review included investigations conducted in different populations that utilized different data collection techniques but yielded similar results across studies.

## Conclusion

This review analyzed the reports on sexual dimorphism in the different odontometric parameters of permanent human dentition using CBCT. Within the scope of this study, it was found that odontometrics of human permanent dentition using CBCT exhibit a certain degree of sexual dimorphism. Both linear and volumetric measurements of teeth can aid sex estimation. The diversity in odontometrics and numerous published reports on odontological SE methods emphasized the need and relevance of SE in human identification. Dimensions of permanent canines show greater variation, especially in mandibular canines, exhibiting the greatest sexual dimorphism. Diverse data sets, parameters evaluated, and methodology followed in these studies included in the review failed to provide concrete evidence to generalize the results. Further studies in various ethnic groups with specific protocols evaluating linear and volumetric measurements of permanent teeth using CBCT are required to verify and validate the findings and strengthen the reliability of this method.

## Supplementary Information

**Additional file 1.** Search strategy. PubMed search strategy.

## Data Availability

The datasets used and/or analysed during the current study are available from the corresponding author on reasonable request.
