# Supplementary material for: Sexual dimorphism in odontometric parameters using cone beam CT: a systematic review
Source: Head Face Med. 2023 Mar 7;19:6. doi: 10.1186/s13005-023-00352-7 (PMC9990232; doi:10.1186/s13005-023-00352-7)
Supplement: Supplementary file 1 — Additional file 1. Search strategy. PubMed search strategy. [file 13005_2023_352_MOESM1_ESM.docx]

**PubMed Search Strategy**

| **Search strategy** | **MeSH/Keywords** | Results |
| --- | --- | --- |
| 1 | (((("CBCT"[All Fields] OR "cone beam computed tomography"[All Fields]) AND "Odontometry"[All Fields]) OR (("odontometric"[All Fields] OR "odontometrical"[All Fields] OR "odontometrics"[All Fields]) AND ("variabilities"[All Fields] OR "variability"[All Fields] OR "variable"[All Fields] OR "variable s"[All Fields] OR "variables"[All Fields] OR "variably"[All Fields])) OR "Teeth"[All Fields] OR "Volumetric assessment"[All Fields] OR (("volum"[All Fields] OR "volume"[All Fields] OR "volumes"[All Fields] OR "voluming"[All Fields]) AND ("teeth s"[All Fields] OR "teeths"[All Fields] OR "tooth"[MeSH Terms] OR "tooth"[All Fields] OR "Teeth"[All Fields] OR "tooth s"[All Fields] OR "tooths"[All Fields])) OR "Volume measurement"[All Fields] OR "Pulp cavity volume"[All Fields] OR "Buccolingual dimension"[All Fields]) AND "Sexual Dimorphism"[All Fields]) OR "Gender assessment"[All Fields] OR "Sex assessment"[All Fields] OR "Sex determination"[All Fields] OR "Gender Determination"[All Fields] OR "Gender prediction"[All Fields] OR (("indicate"[All Fields] OR "indicated"[All Fields] OR "indicates"[All Fields] OR "indicating"[All Fields] OR "indicative"[All Fields] OR "indicatives"[All Fields] OR "indicators and reagents"[Pharmacological Action] OR "indicators and reagents"[MeSH Terms] OR ("indicators"[All Fields] AND "reagents"[All Fields]) OR "indicators and reagents"[All Fields] OR "indicator"[All Fields] OR "indicators"[All Fields] OR "indice"[All Fields] OR "indices"[All Fields]) AND ("sex"[MeSH Terms] OR "sex"[All Fields])) OR "Sex estimation"[All Fields] | 1,53,343 |
| 2 | (((("CBCT"[All Fields] OR "cone beam computed tomography"[All Fields]) AND "Odontometry"[All Fields]) OR "Teeth"[All Fields] OR "Volumetric assessment"[All Fields] OR "Volume measurement"[All Fields] OR "Pulp cavity volume"[All Fields] OR "Buccolingual dimension"[All Fields]) AND "Sexual Dimorphism"[All Fields]) OR "Gender assessment"[All Fields] OR "Sex assessment"[All Fields] OR "Sex determination"[All Fields] OR "Gender Determination"[All Fields] OR "Gender prediction"[All Fields] OR "Sex estimation"[All Fields] | 14,302 |
| 3 | ((((("CBCT"[All Fields] OR "cone beam computed tomography"[All Fields]) AND "Odontometry"[All Fields]) OR "Teeth"[All Fields] OR "Volumetric assessment"[All Fields] OR "Volume measurement"[All Fields] OR "Pulp cavity volume"[All Fields] OR "Buccolingual dimension"[All Fields]) AND "Sexual Dimorphism"[All Fields]) OR "Gender assessment"[All Fields] OR "Sex assessment"[All Fields] OR "Sex determination"[All Fields] OR "Gender Determination"[All Fields] OR "Gender prediction"[All Fields] OR "Sex estimation"[All Fields]) AND (2000:2022[pdat]) | 9,363 |
| 4 | ((((("CBCT"[All Fields] OR "cone beam computed tomography"[All Fields]) AND "Odontometry"[All Fields]) OR "Teeth"[All Fields] OR "Volumetric assessment"[All Fields] OR "Volume measurement"[All Fields] OR "Pulp cavity volume"[All Fields] OR "Buccolingual dimension"[All Fields]) AND "Sexual Dimorphism"[All Fields]) OR "Gender assessment"[All Fields] OR "Sex assessment"[All Fields] OR "Sex determination"[All Fields] OR "Gender Determination"[All Fields] OR "Gender prediction"[All Fields] OR "Sex estimation"[All Fields]) AND ((humans[Filter]) AND (2000:2022[pdat])) | 3,248 |
| 5 | ((((("CBCT"[All Fields] OR "cone beam computed tomography"[All Fields]) AND "Odontometry"[All Fields]) OR "Teeth"[All Fields] OR "Volumetric assessment"[All Fields] OR "Volume measurement"[All Fields] OR "Pulp cavity volume"[All Fields] OR "Buccolingual dimension"[All Fields]) AND "Sexual Dimorphism"[All Fields]) OR "Gender assessment"[All Fields] OR "Sex assessment"[All Fields] OR "Sex determination"[All Fields] OR "Gender Determination"[All Fields] OR "Gender prediction"[All Fields] OR "Sex estimation"[All Fields]) AND ((humans[Filter]) AND (english[Filter]) AND (2000:2022[pdat])) | 3,076 |
| 6 | Title and abstract screening | 3062 |
| 7 | Full text with inclusion and exclusion criteria | 12 |
| 8 | Excluded studies | 3050 |
